# Openness, inclusion and transparency in the practice of public involvement in research: A reflective exercise to develop best practice recommendations

**DOI:** 10.1111/hex.12609

**Published:** 2017-11-03

**Authors:** Laura J.E. Brown, Tommy Dickinson, Stuart Smith, Christine Brown Wilson, Maria Horne, Kate Torkington, Paul Simpson

**Affiliations:** ^1^ Division of Psychology and Mental Health University of Manchester Manchester UK; ^2^ Department of Mental Health Nursing Florence Nightingale Faculty of Nursing and Midwifery King's College London London UK; ^3^ Community Representative in the Older People's Understanding of Sexuality (OPUS) Research Team Manchester UK; ^4^ School of Nursing, Midwifery and Social work University of Queensland Brisbane QLD Australia; ^5^ School of Healthcare University of Leeds Leeds UK; ^6^ Department of Applied Health and Social Care Edge Hill University Ormskirk UK

**Keywords:** communication, community, intimacy and sexuality, productivity, resources, roles

## Abstract

**Context:**

Reflective accounts of public involvement in research (PI) are important for helping researchers plan and deliver more effective PI activities. In particular, there is a need to address power differentials between team members that can prohibit effective and meaningful involvement.

**Objective:**

To critically reflect on the PI practices that underpinned our research project on intimacy and sexuality in care homes, to develop a series of recommendations for improving future PI activities.

**Setting:**

The research team comprised five academics from nursing, public health, sociology and psychology, and two members of the public with experience of sex education, and lesbian, gay, bisexual and trans issues in older populations. In order to address power differentials within the group, we developed an approach to PI practice that was grounded in values of openness, inclusion and transparency.

**Method:**

Reflective commentaries on the strengths and weaknesses of the team's approach to PI were gathered through interviews and open‐ended questionnaires with research team members. These views were collated and discussed at a workshop comprising research team members and an additional member of the public to generate recommendations for future PI practice.

**Results:**

A number of strengths and limitations of our approach to PI were identified. Clear recommendations for improving PI practice were developed for three broad areas of identified difficulty: (i) communication within and between meetings; (ii) the roles and responsibilities of team members; and (iii) PI resources and productivity.

**Discussion and conclusion:**

These recommendations add to the developing body of guidance for conducting effective PI.

## 1 INTRODUCTION

Public involvement in research (PI) is defined as research that is “carried out ‘with’ or ‘by’ members of the public rather than ‘to’, ‘about’ or ‘for’ them.”^1^ Members of the public involved in research are often drawn from specific target populations, such as patients, service users, or those with a shared experience or demographic characteristic, such as age group.^2^ PI is increasingly recognized as being an important element of health and social research, with advocates outlining its role in supporting values such as empowerment and increased transparency,^2^ as well as the benefits arising from the unique knowledge and insights that members of the public can bring to research.^3^

Various guidelines exist that provide advice to researchers on how to conduct effective PI. These include a comprehensive set of briefing notes from the national advisory group on service user involvement “INVOLVE,”^1^ as well as a “how‐to” guide for health and biomedical researchers.^4^ These resources are supplemented by specific insights that have emerged from reflective exercises undertaken by researchers and members of the public on the PI practices that they have experienced. For instance, a reflective report by academics and patients working together in rheumatology research^5^ revealed specific examples of typical academic working practices that could hinder effective communication in PI projects. These included the chance “corridor” meetings in the workplace from which members of the public are excluded, as well as the pace and last‐minute nature that is typical of academic decision making. In addition, an ethnographic study of three health‐focused PI case studies reported how opportunities for less formal discussion between team members (such as phone calls and shared travel time) can be useful for enabling public members of the team to contribute their thoughts outside of structured meetings.^6^

Insights from reflective studies can also make valuable contributions to debates around best practice in PI. For instance, whilst many researchers have emphasized the importance of providing clear role descriptions in PI,^6^, ^7^, ^8^ there are more mixed views as to whether members of the public should be given training in the roles they undertake. Some consider this to be essential to effective PI, whilst others argue that it is unreasonable to assume that somebody with little or no research background would be able to master the higher levels of skill and understanding required for some research‐related tasks.^9^ Moreover, the idea of training members of the public in research methods could also be considered incompatible with the underpinning principle of valuing members of the public precisely *because* of the different mindsets, skills and expertise that they bring to a research team.^3^ Reflective studies that specifically consider the appropriateness of roles and training for members of the public within PI are therefore needed in order to inform this debate.

Many of the pervasive issues associated with PI, such as communication difficulties, and the nature of roles undertaken, relate to the power relationships that exist between members of the public and academic clinical or academic researchers. Such issues could be exacerbated by the internal power hierarchies that often exist within health and social care settings,^10^ and which risk further inhibiting effective teamwork when involving the public in multidisciplinary research teams. Whilst there is clear recognition of the benefits of creating a non‐hierarchical team structure, in which the contributions of all team members are equally valued,^6^, ^11^ successfully achieving such a flat power structure therefore remains a challenge. Indeed, even when academic members have the best intentions of eliminating power differentials, their desire to protect members of the public, for instance by minimizing burden, challenge or stress, may inadvertently contribute to power imbalances. For instance, researchers working with members of breast cancer self‐help groups described how they were accused of being unconsciously paternalistic by a reviewer because they had not considered it appropriate to involve the self‐help group members in the analysis of data.^9^ Again, reflective reports from PI projects that have attempted to address issues of power and inclusion would therefore be useful for developing practical guidance on how the balance between meaningful, yet manageable, involvement can be achieved.

To further develop knowledge and guidance on effective PI practice, we conducted a reflective exercise on a recent PI project undertaken by our research team. The OPUS (Older People’s Understanding of Sexuality) research team was initially established by four academics and a clinical research officer, to conduct a consultative research project on understanding issues of sexuality and intimacy in care homes for older people.^12^ Following an award of funding, the research team was expanded to include an additional academic researcher, and two older members of the public (referred to hereon in as community representatives), one male, and one female, who were recruited through contact with a City Council‐run volunteer forum for improving the lives of Manchester's older citizens, and a lesbian, gay, bisexual and trans (LGB&T) group run by a local branch of the charity “Age UK”. Both community representatives were of White British ethnicity. One was aged 81 years, was educated to degree level and had previous experience of working in sex education. The other was aged 74 years and had been employed in a diverse range of service and clerical occupations. When recruiting community representatives, the research team made it clear that no particular academic knowledge was required for the role but that volunteers should feel confident about expressing insights and opinions on sex, intimacy and sexual difference as an older individual.

In order to identify effective ways of minimizing power differentials, and promoting meaningful and appropriate involvement, we developed an approach to PI practice that was grounded in principles of openness, inclusion and transparency. The reflective exercise was conducted towards the end of the research project, and aimed to probe perceptions of how well the approach to PI had worked, and to identify specific recommendations through which future PI practices could be improved. In this study, we describe the model of PI practice that was used in the OPUS study, and the methods and outcomes of the reflective exercise, to add new insights into practices that optimize effective and meaningful PI involvement.

## 2 PRACTICES WITHIN THE OPUS PROJECT

In an attempt to minimize power differentials within our research team, we ensured that community representatives were invited to be involved with all aspects of the project for which specific training was not considered necessary. Specifically, they did not participate in recruitment, data collection or formal data analysis, but were included in discussions about plans for recruitment, themes arising from primary data (interview and focus group transcripts), changes to the study plan, the writing up and broader dissemination of study findings, and future grant opportunities. Rather than developing formal role descriptions at the start of the project, community members’ roles were instead allowed to develop and evolve naturally throughout the project.

In order to prevent the community representatives being excluded from key discussions, and to minimize a sense of hierarchy within the group, they were invited to all research group meetings and were given hard copies of all meeting agendas and minutes. As some academic members of the team were separated over large geographical distances, some meetings involved the use of Skype. Both community representatives also participated as speakers and discussants with the academic team members at a half‐day conference that was held part‐way through the study, at the University of Manchester, to share initial findings and gather reflections on the underlying research aims. One of the community representatives also spoke about the work at an additional academic conference, at another local University. In line with advice from previous reflective exercises,^6^ we also provided considerable opportunity for less formal interaction between team members. This included informal social meetings between subsets of the team in public places (such as cafes), as well as the adoption of an informal tone before and during team meetings. The principal investigator of the project also had additional telephone conversations with the community representatives throughout the project period.

In addition to meetings, some discussion and sharing of information were performed through email. One of the community representatives was an email user, and so was included in the majority of group email communication between team members, including many email discussions relating to arrangements for future meetings; discussions of ethics amendments and reporting; plans for future funding, collaboration, impact and dissemination; and feedback from external meetings and conferences attended. Drafts of key documents, including summaries of interim results for dissemination, and some conference slides, were also shared by email, with opportunities for comment solicited. In an attempt to balance inclusivity with manageability and burden, the community representative was copied into most, but not all emails. No decisions were made a priori about which emails the community representative should be copied into. Rather, decisions regarding who to include in emails were made by individual team members at the point at which they wrote the email. The team regularly discussed these decisions during meetings in order to adhere to our values of openness, inclusion and transparency. Generally, the community representative did not receive emails that related to the more technical aspects of the project, such as project finances; specific challenges and opportunities relating to the recruitment of individual care homes into the project; the writing of a literature review; and networking and future funding opportunities. The second community representative was not an email user, and so was not included in any of the email conversation. However, in line with our values of openness, inclusion and transparency, hard copies of key study information (including study documents scrutinized and approved by the ethics committee), as well as transcripts of raw data and field notes taken from the half‐day conference, were sent to both community representatives through the post.

## 3 REFLECTIVE EXERCISE

Feedback on the effectiveness of our PI practice was gathered through a series of structured activities. First, each community representative was interviewed individually by one or two of the academic team members, about their thoughts and feelings regarding their involvement with the project, including what might have improved the process. In one instance, this was in the presence of a new, community representative, who was known personally by one of the academic team members, but was external to the OPUS team. This community representative was male, aged 56 years and educated to “O”‐level, with a background in magazine production. These conversations were audio‐recorded, and key points extracted from the notes and transcripts. Each academic member of the team also provided written responses to five open‐ended questions that probed their perspectives on the benefits and disadvantages to involving the community representatives in the research; what did and did not work well about the ways that they were involved; and how the PI practices could have been improved. These notes and responses were collated by the lead author and organized into groups of common views.

The groups of common views were then presented and discussed at a workshop attended by three members of the academic team (authors LJEB, PS and TD), one of the two OPUS community representatives (author SS), as well as the new, external male community representative, who was asked to attend in place of one of the original community representatives, who was not in a position to participate in the project at that time. The purpose of the workshop was to generate a set of recommendations for effective PI that was based on the group's experience with the OPUS project. It was facilitated by one of the academic team members (LJEB) and involved all five attendees, as a group, systematically discussing each of the positive aspects, issues and suggestions for improvements that had been collated prior to the workshop, and then agreeing as a group on a list of recommendations for future practice. Drafts of the reflections and recommendations were then shared with the academic team and one of the OPUS community representatives and modified through feedback.

## 4 REFLECTIONS AND RECOMMENDATIONS

### 4.1 Positive aspects

Members of the team reported a number of positive aspects associated with the PI practice. In particular, team members spoke of the enjoyment that came from working together, with one of the community representatives stating that the informal social events provided a “real good laugh.” Academic members also mentioned the “comedic element” that one of the representatives brought to the meetings, which “changed the dynamic in a positive way,” and “made the meetings more enjoyable.” One of the community members also stressed how welcome they felt at the start of the project, and how their level of comfort increased as working relationships developed.

Another key benefit was the perceived sense of “authenticity” and “credibility” that the academics felt was added to the study's results by including older community representatives in the team. This was particularly the case when the team presented their findings at the half‐day conference, with one academic reflecting how the community representatives were “very good at getting across the range of needs and views around sex, sexuality, intimacy and inclusion in service provision,” and that their contributions would have been more likely to “resonate with older people.”

There was also clear recognition by both the academics and community representatives of the unique contributions that the community representatives made to the project, with recognition that they “asked pertinent questions” that the academics “may never have thought of” and pushed the researchers “to expand on certain issues” and to clarify some of the terminology used. In addition, academic team members reported that the community representatives kept the team “grounded” by pointing out when they “hadn't defined a term” or “had over‐interpreted something” and sometimes offered alternative explanations of participants’ responses.

### 4.2 Areas for improvement

A number of areas for improvement were also identified. With regard to group discussions, both academic and community representatives recognized that there were times when the community representatives could not understand or follow what was being discussed. This was described as often being due to the “esoteric” and “academic” topics that needed to be discussed (e.g. ethics and funding applications), the use of academic jargon and the rapid pace of discussion. Communication involving Skype was also made more frustrating by technical problems that affected the meeting. Community representatives also reported that they would have liked more time and opportunity to clarify things that they did not understand, particularly as they sometimes felt “self‐conscious” about indicating a lack of understanding, and that interrupting a conversation was felt to be “bad manners.” One community team member also stated that it was sometimes difficult to contribute to discussions because “I might have thought about mentioning something but then by the time I've got the opportunity, I've forgotten.” Academic team members also felt that “subtle power dynamics and a sense of social obligation” may also have inhibited community representatives’ inclination to voice concerns during meetings.

The type and amount of project information that community members were given, or asked to discuss, was also felt to be inappropriate at times. For one thing, the community representatives were not always able to understand the purpose or content of some of the printed information that they were sent in the post and said that it did not always feel “relevant” to them or their skill sets. For instance, one of the community representatives spoke about how, although able to understand what participants were saying in the interview transcripts that were sent out (and, indeed, sometimes offered alternative explanations for responses), the participants sometimes spoke “about something and nothing,” which made it “very difficult to understand what it is they are trying to say” in relation to the aims of the project. One of the academics also felt that sharing research data was not “particularly helpful” as the community representatives did not have “experience of research and so were passing personal comments.” An academic team member also reflected that it felt “unfair” to ask community representatives to “make the effort to come in for a meeting when they were then left listening to parts of a conversation that were not relevant to them, or that they were not able to contribute to,” and that the community representative who used email was included “into too many emails that he didn't need to see.”’ One academic also reflected that more opportunities could have been given to the community representatives to “tell us about their worlds” rather than being so “business‐oriented.” All members of the team also recognized that the amount of information posted out to community representatives was too great, and perhaps overwhelming. The difficulties of being able to keep so much information organized were also pointed out, with one community member describing how the material would go “in a pile and things get shoved on top of it.”

Other issues, which were predominantly raised by academic team members, related to levels of efficiency and productivity being lower than what they were used to. In addition to the “extra effort” needed in terms of “organizing meetings and general logistics,” the strategy of including community representatives in all meetings led to a sense that “the academic talk had to be limited because we were aware of not wanting to use jargon” and that sometimes the conversation had become more technical because “there were no other opportunities to be able to discuss this with the team.” The academic team's desire to maintain a friendly, inclusive and enjoyable atmosphere also meant that some academic team members felt that meetings were not always as “efficient” or “productive” as they were used to, although the “positives” of involving community representatives were also very much recognized.

### 4.3 Recommendations for future practice

A set of recommendations for addressing the common issues identified by team members was developed through discussion at the workshop and later refined through iterative team feedback. These are presented in Box 1 and grouped into the broad domains of Communication, Roles and Responsibilities, and Resources and Productivity.

Box 1 **Recommendations for improving PPI activities**

**Communication**

- Ground rules for communication in meetings should be collectively agreed at the start of the project, and periodically revisited. These might include procedures that enable team members to easily indicate that they do not understand or wish to say something (such as raising up their hand or a coloured card), or period checks as to whether community representatives wish to contribute or clarify anything.
- Community representatives should be given pens and paper during meetings, and encouraged to note down thoughts that come to them.
- A glossary of common terms (used by both academics and community representatives) could be built up through discussions, and used in meetings, to help team members to understand one another's jargon.
- Agreement on the type, amount, and format of information sent to community representatives between meetings (including email conversations) should be agreed on an individual basis, and periodically reviewed.
- A ‘core plus’ approach to information sent between meetings could be considered, in which all community representatives are given a core minimum of information, but with all other information made easily available, as desired.
- If providing long documents (e.g. interview transcripts) it may be useful to provide summaries of key points, and clear cover letters that outline why the information has been sent, and what they are expected to do with it. Community representatives should also be reminded that they can challenge the summaries if they feel that important aspects from the longer documents have been omitted or misrepresented.
- Specific social meetings should be organised between academics and community representatives to break down social barriers and increase approachability.

**Roles and Responsibilities**

- Role descriptions that outline the type and extent of community representatives’ involvement can be helpful for agreeing and communicating expectations. Such role descriptions should be periodically reviewed and updated, as necessary.
- A larger team of community representatives could be involved in the project (e.g. at different times for different roles, or just to replace members who become less available) so that the sense of burden/responsibility is reduced.
- If community representatives are interested in developing their skills, then relevant training could be provided externally or by the research team.
- Separate meetings should be organised specifically for academic business, such as grant funding, journal selection etc. These meetings should be *open* to community representatives, and yet made clear that the information discussed is unlikely to be *relevant* to them. To maintain transparency and inclusivity, agendas for these meetings should be circulated in advance, and minutes made available afterwards.

**Resources and Productivity**

- Clear details of a meeting's objectives and agenda should be circulated in advance.
- Community representatives should be offered folders to help them to store and organise printed information generated by the study.
•It may be useful to have a designated member of the team (or independent person) who community representatives could approach if they have any concerns about their involvement that they do not feel comfortable raising with the wider team.
- Sufficient time for the additional administration and meeting time required to do PI properly should be planned and budgeted for early on.

## 5 DISCUSSION

Our aim within the OPUS team was to foster an approach to PI that was open, inclusive and transparent. By gathering each team member's reflections on how well the PI approach worked and then discussing these in a solution‐focussed workshop, we identified various strengths and limitations of our working practices and generated recommendations as to how PI practice could be improved in the future. These issues and recommendations related to themes of communication; roles and responsibilities; and resources, which are commonly reported as being problematic in PI studies, thus providing relevant guidance that is applicable to a range of health and social research.

As with other reflective PI studies,^5^, ^6^ effective communication (across a variety of mediums) was challenging for our research team. For one thing, our philosophy of openness, inclusion and transparency led to community representatives feeling overwhelmed by the volume of written information that was sent and also a little confused as to what they were expected to do with all the information that was sent. Some academic members of the team also reflected on the feeling that, in an attempt to uphold this philosophy, they ended up overburdening the community representatives by over‐including them in discussions of topics that were of little interest or relevance to them. The suggestion of a “core plus” approach to information, along with summaries of key documents, and signposting to explain why the information has been sent, therefore seemed like an appropriate compromise between openness and manageability. The recommendation to agree, and regularly review, the type, amount, and format of communication between meetings (including the appropriateness, and level, of email communication) should also help to ensure that the appropriate balance between inclusivity and burden can be found for each individual.

Despite efforts within our team to reduce power differentials and a sense of hierarchy, one of the community representatives reported finding it difficult to contribute to some team discussions, particularly when this required interrupting the flow of discussion. Previous good practice PI guidelines have emphasized the importance of regularly checking PI team members’ understanding of the discussion and soliciting their contributions.^1^ However, a reflection in our study was that community representatives sometimes struggled to remember points they wanted to make, and so would not always be able to contribute these if solicited later in the discussion. Therefore, our findings suggest that ground rules for communication should also include easy ways for PI members to interrupt conversations (eg by using hand gestures) and also (where appropriate) support the use of note‐taking materials to record ideas or points that they can contribute at a later time point. The value of periodically reviewing communication strategies within the team, and adjusting as necessary, was also recognized by team members.

Previous reflective reports^5^, ^6^, ^7^ and PI guidelines^1^, ^4^ have recommended the use of developing and providing role descriptions for PI members. In our study, we tried an alternative approach of role development, in which community representatives were encouraged to be involved in any aspect of the project that felt appropriate without formal training, allowing roles to evolve more naturally within the team. Through our reflective process, we identified some problems with this approach, such as a lack of clarity about what was expected of the community representatives, as well as concerns around some topics or tasks feeling irrelevant or inappropriate for them to be involved with. Recommendations emerging from our study therefore reinforce the value of the more conventional approach of formalizing individuals’ roles through negotiated role descriptions.^6^, ^7^, ^8^

With regard to debates around training for PI members, the key message emerging from our study was that training opportunities should be made available if PI members express a personal interest in developing their own skills, rather than as a requisite for taking part. These recommendations therefore reflect the principle of valuing members of the public precisely *because of* the skills, expertise and perspectives that they come with,^3^ rather than attempting to train them to develop the skills, expertise and perspectives that are already held by the academic members of the team. The recommendation that separate meetings be organized for the discussion of specific academic issues (such as funding and publication plans) could also be seen as a way of recognizing, respecting and supporting the respective contributions that each “type” of team member can bring to discussions. Separating more technical topics from general meetings could also help to minimize the power differentials that can be created when community members become excluded from discussions due to their lack of expertise on a certain topic.^13^

A final set of issues and recommendations that emerged from this project related to the additional resources that are needed to enable successful PI. Others have emphasized the need to budget for additional time and money when planning PI work,^4^, ^7^ and INVOLVE have even developed a cost calculator for this purpose.^14^ However, not all of the cost and resource implications for effective PPI have been fully articulated. Here, we add to the literature by detailing some of these additional costs, and the reasons for them. For instance, although “small talk” within meetings was perceived to be enjoyable and useful for building relationships, some academic members of the team reported some frustrations relating to perceptions of reduced efficiency and productivity that this informality added. Explicitly planning for such rapport‐developing opportunities outside of key meetings could therefore help to reduce some of the frustrations experienced. Other resources that our study suggested should be planned and budgeted for include: additional meeting time so that more technical topics can be discussed separate from core PI meetings; administrative support for summarizing key documents, and writing covering letters to accompany them; time for the negotiation (and revision) of roles and communication strategies; and buying items of stationary to help community representatives to contribute to discussions and organize study materials. As others^1^ have previously recommended, having a designated person (perhaps external to the team) who community representatives can approach if they are unhappy with any aspect of the project, was also seen as a useful resource that should be budgeted for.

## 6 CONCLUSION

This reflective exercise proved useful for identifying new recommendations for increasing the effectiveness of PI practice. In particular, we show how principles of openness, inclusion and transparency can be implemented in PI practice but that they must be balanced against issues of burden and must respect the differences between academic researchers and members of the public. We acknowledge that power differentials in research contexts are inevitable, sometimes fluid and will never be fully equalized, but there are good ethical and professional reasons for working with community representatives to minimize them, or use them productively. The recommendations emerging from this study add to a growing body of useful, reflective literature designed to increase the effectiveness of PI practice, and which are of relevance to a broad range of health and social researchers. As such recommendations are based on the subjective views and experiences of small numbers of people, future work that tests the effectiveness of specific recommendations, as well as their applicability to specific types of projects and research teams, could be a useful next step forward in creating robust, evidence‐based guidelines for PI.

## CONFLICTS OF INTEREST

The authors report there to be no conflicts.
